# Synergistic Regulation of Phase and Nanostructure of Nickel Molybdate for Enhanced Supercapacitor Performance

**DOI:** 10.3390/nano14221798

**Published:** 2024-11-08

**Authors:** Yining Wang, Yuhan Cui, Yue Song, Chen Zhou

**Affiliations:** 1 School of Chemistry and Environmental Engineering, Changchun University of Science and Technology, Changchun 130022, China; 2023200212@mails.cust.edu.cn (Y.C.); 2024101476@mails.cust.edu.cn (Y.S.); 2 Jilin Provincial International Joint Research Center of Photo-Functional Materials and Chemistry, Changchun 130022, China; zhouchen@cust.edu.cn

**Keywords:** nickel molybdate, surface morphology, hybrid supercapacitors, battery-type electrode

## Abstract

Nickel molybdate, which has a relatively high theoretical capacity, demonstrates potential for use in supercapacitors. However, its inferior electrical conductivity and cycling stability have led to poor electrochemical performance. Nanostructure engineering of NiMoO_4_ is an efficient strategy to overcome its performance limitations as an electrode. Here, a facile approach is reported for the precise phase regulation and nanostructure of NiMoO_4_ by manipulating the synthesis parameters, including duration, precursor selection, and urea concentration. The electrochemical properties of the electrode materials are also investigated. It is interesting to note that the β-NiMoO_4_ nanosheets show a decent specific capacity of 332.8 C/g at 1 A/g, surpassing the 252.6 C/g of the α-NiMoO_4_ nanorods. Furthermore, the supercapacitor device constructed with β-NiMoO_4_ and reduced graphene oxide hydrogel (rGH) electrodes achieves an acceptable energy density of 36.1 Wh kg^−1^, while retaining 70.2% after 5000 cycles.

## 1. Introduction

In light of the concurrent issues posed by the global energy crisis and environmental degradation, the advancement of effective and sustainable energy storage technologies has become crucial [1,2]. Supercapacitors have emerged as a focal point of research in the field of power and energy owing to their high-power density, extended cycle life, and rapid charging and discharging capabilities [3,4]. This type of energy storage technology, positioned between conventional capacitors and secondary batteries, exhibits significant potential for application in sectors such as renewable energy vehicles, electronic devices, and telecommunications equipment, due to its environmentally friendly, efficient, and dependable attributes [5,6,7]. The swift advancement of portable electronic devices and electric vehicles has imposed elevated demands on the energy density and power density of supercapacitors, thus making it essential to innovate new high-performance electrode materials.

The ideal electrode material ought to possess an extensive specific surface area, minimal internal resistance, good wettability, and excellent chemical stability [8]. Among a variety of electrode materials, transition metal oxides (TMOs) have garnered significant interest because of their elevated specific capacitance and environmental friendliness [9,10]. Due to the presence of variable valence metal elements, transition metal oxides achieve efficient energy storage through highly reversible redox reactions [11,12]. In addition, bimetallic oxides exhibit superior electrochemical performance when contrasted with monometallic oxides, attributed to their increased number of active sites and superior conductivity [13].

Nickel molybdate, as a transition metal oxide, has demonstrated significant promise in the domain of supercapacitors due to its simple preparation and eco-friendly characteristics [14]. Despite the high theoretical capacity of nickel molybdate, the poor electrical conductivity limits the full realization of its electrochemical properties [15]. Therefore, optimizing the electrochemical performance of nickel molybdate electrodes is of great importance to meet the growing demand for energy storage. Recently, researchers have effectively improved the electrochemical activity and electron transport efficiency of nickel molybdate by designing different nanostructures, such as nanorods, [16] nanowires [17], nanoparticles [18], nanosheets [19], and nanoflowers [20]. In addition, the combination of other active materials with nickel molybdate to form composites [21,22,23] or multi-dimensional core-shell structured materials [24,25,26,27] is also an effective strategy to improve its electrochemical properties. It is worth noting that several studies have confirmed the possibility of synthesizing α-NiMoO_4_ and β-NiMoO_4_ at room temperature [28]. For example, Ratha et al. found that despite having the same monoclinic crystal structure, α-NiMoO_4_ and β-NiMoO_4_ varied greatly in their electronic structure, chemical environment, and structural characteristics because of variations in the ionic coordination of Mo^6+^ [29]. Naik et al. discovered that β-NiMoO_4_ with a single ionization vacancy was better suited for electrochemical applications than α-NiMoO_4_ due to its higher intrinsic catalytic activity and electrochemical properties [30]. However, in contrast to the wide application of α-NiMoO_4_, the research of β-NiMoO_4_ in energy storage/conversion applications is obviously insufficient [31]. Moreover, the existing studies still have shortcomings in the crystal phase regulation and nanostructure design of nickel molybdate. The precise regulation of phases is essential for optimizing the electronic structure and ion diffusion paths of nickel molybdate, whereas the strategic engineering of nanostructures can facilitate the augmentation of the specific surface area and the availability of active sites [32,33]. As a result, in order to guide the rational design of novel NiMoO_4_-based electrode materials, it is crucial to clarify the role of synthesis variables such as duration, precursor selection, and urea. This not only enables us to obtain a better understanding of the crystal transformation mechanism of nickel molybdate, but it also allows for the dual optimization of phase regulation and structure of nickel molybdate, promoting its application in the field of energy storage/conversion.

The choice and optimization of negative electrode materials is another important step in the design of supercapacitors, and it has a direct impact on the devices’ performance, cost, and possibility for market use. Carbon materials, as one of the first electrode materials widely employed in the energy storage field, have received a lot of attention because of their significant environmental protection value and broad application prospects [34,35]. Graphene stands out among carbon materials due to its adjustable large specific surface area (~2600 m^2^/g), excellent chemical stability, and extremely high mechanical strength (1 Tpa) [36]. However, graphene nanosheets tend to aggregate due to π–π interactions and van der Waals pressures, resulting in a considerable drop in their specific surface area and capacitance when applied [37]. With the recent successful preparation of 3D reduced graphene oxide hydrogel (rGH) materials, the method may effectively restrict the aggregation of graphene sheets, hence addressing the above issues [38]. Moreover, the electrochemical performance of the material significantly improves due to the rich pore characteristics of its three-dimensional structure, which facilitates the movement of ions and electrons [39,40].

In this work, a strategy for regulating the phase and nanostructure of nickel molybdate has been reported. The precise adjustment of α-NiMoO_4_ nanorods and β-NiMoO_4_ nanosheets was achieved by adjusting the synthetic variables. As a result, the synthesized β-NiMoO_4_ electrode showed a specific capacity of 363.6 C/g at 0.5 A/g, along with commendable cycling stability. Moreover, the assembled ASC exhibited a voltage window of 1.6 V and a specific capacity of 162.4 C/g at 0.5 A/g. This strategy could offer novel insights for the advancement of other high-efficiency electrode materials.

## 2. Materials and Methods

### 2.1. Chemicals

Nickel (II) acetate tetrahydrate (Ni(CH_3_COO)_2_·4H_2_O, 99%), nickel nitrate hexahydrate (Ni(NO_3_)_2_·6H_2_O, 98%), hydrochloric acid (HCl, 36%), urea (CO(NH_2_)_2_, 99%), ammonia (NH_3_·H_2_O, 35%), potassium hydroxide (KOH, 90%), ammonium molybdate ((NH_4_)_6_Mo_7_O_24_·4H_2_O, 99%), sodium molybdate dihydrate (Na_2_MoO_4_·2H_2_O, 99.9%), and hydrazine hydrate (N_2_H_4_·H_2_O, 85%) were purchased from Sinopharm Chemical Reagent Co., Ltd. (Nanjing, China). Polytetrafluoroethylene (PTFE, 60%) and carbon black were acquired from Saibo (Shanghai, China). All chemicals were utilized in their original state, requiring no additional purification. Solutions in this work were meticulously prepared using deionized (DI) water, which was produced by a Milli-Q water purification system, ensuring that the experiments were conducted with the requisite level of purity.

### 2.2. Materials Synthesis

#### 2.2.1. Synthesis of α-NiMoO_4_

Nickel (II) acetate tetrahydrate (3 mmol) and ammonium molybdate (3 mmol) were dissolved in deionized water (40 mL). Following a stirring period of 2 h, the homogeneous mixture was sealed in an autoclave and maintained at 150 °C for 10 h. Upon completion of the reaction, the precursor was obtained by centrifugation, washing, and drying. Then, the precursor was annealed 500 °C at 2 °C/min under an air atmosphere for 4 h. The rod-like α-NiMoO_4_ was finally obtained.

#### 2.2.2. Synthesis of β-NiMoO_4_

First, 3 mmol of nickel (II) acetate tetrahydrate was mixed with 3 mmol ammonium molybdate without solvent, and then an aqueous urea solution with a concentration of 4 M (10 mL) was introduced into the aforementioned mixture. After a 2 h stirring period to ensure homogeneity, the mixture was sealed within an autoclave and reacted at 150 °C for 2 h to ~10 h. Upon the conclusion of the reaction, the precursor was obtained by centrifugation, washing, and drying. Then, the precursor was annealed at 500 °C at 2 °C/min under an air atmosphere for 4 h. The samples are designated as NiMoO_4_-2H, NiMoO_4_-4H, NiMoO_4_-6H, and NiMoO_4_-10H. Following the same process, a series of NiMoO_4_ samples were synthesized by incorporating varying concentrations of urea, resulting in the creation of NiMoO_4_-0M, NiMoO_4_-1M, NiMoO_4_-2M, and NiMoO_4_-4M. Furthermore, the NiMoO_4_ samples (designated as NNSM, NASM, and NAAM) were also prepared in controlled experiments with different solvents to illustrate the effect of solvent variation on its morphology and structure.

#### 2.2.3. Synthesis of rGH

The rGH was synthesized following the procedures detailed in our previous research [41]. In brief, the synthesis of graphite oxide (GO) was carried out following the Hummers method in the literature [42]. Add 100 μL of 30 wt% hydrazine hydrate solution to the appropriate amount of GO solution at a concentration of 2 mg/mL. Subsequently, 150 µL of the above dispersion was pipetted individually into small customized glass tubes, and then these tubes were transferred to a 100 mL autoclave and subjected to a heat treatment at 180 °C for 12 h. These glass tubes were taken out of the autoclave after they had heated, and the hydrogel products obtained from the glass tubes were washed and soaked repeatedly with DI water for further use.

### 2.3. Characterization

Field-emission scanning electron microscopy (FESEM, FEI Quanta 250F, FEI Ltd., Shanghai, China) and transmission electron microscopy (TEM, JEOL JEM-2100, JEOL Ltd., Tokyo, Japan) were utilized to examine the micromorphology of the obtained products. Sample crystal structures were analyzed using an X-ray diffractometer (XRD, Bruker D8 Advance, Bruker, Billerica, MA, USA). The Raman spectra were acquired utilizing a Raman spectrometer (Raman, Thermo Scientific DXR3, Waltham, MA, USA), which was equipped with an argon ion laser featuring an excitation wavelength of 532 nm.

### 2.4. Electrochemical Measurements

All electrochemical tests in 3 M KOH were conducted using a VMP3 workstation (Biologic, Seyssinet-Pariset, France). To prepare the working electrode, activated material was usually mixed with carbon black and polytetrafluoroethylene (PTFE) in an 8:1:1 weight ratio before being painted onto the nickel foam. After drying in a vacuum oven at 60 °C for 6 h, the electrode was immersed in a 3 M KOH electrolyte before use. The Pt electrode was used as the corresponding counter electrode, while the commercial saturated calomel electrode (SCE) was chosen as the reference. Battery electrochemical examination (LAND CT 2001A, Wuhan, China) was selected to measure cycling stability, and EIS measurement was performed at frequencies ranging from 0.01 Hz to 10^6^ Hz with 5 mV sinusoidal voltage. The 2032-coin cells were assembled with NiMoO_4_ serving as the positive electrode, rGH as the negative electrode, and a micro-porous membrane to separate the two electrodes.

## 3. Results and Discussion

### 3.1. Synthesis and Characterization of NiMoO_4_

**Figure 1:**
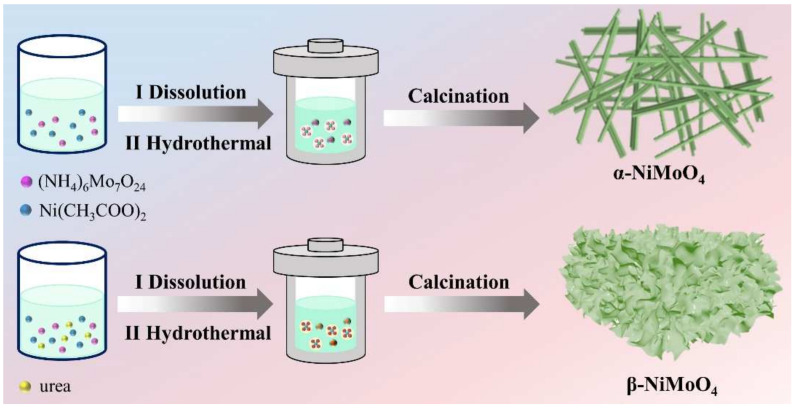
Diagrammatic representation of NiMoO_4_ synthesis process.

Figure 1 shows the process for preparation of α-NiMoO_4_ and β-NiMoO_4_. It was found that urea, as an effective additive, not only can modulate the morphology of the samples but also can induce their phase transformation. It is important to understand the mechanism of action of urea in the process of hydrothermal synthesis and optimize the synthesis conditions of materials.

**Figure 2:**
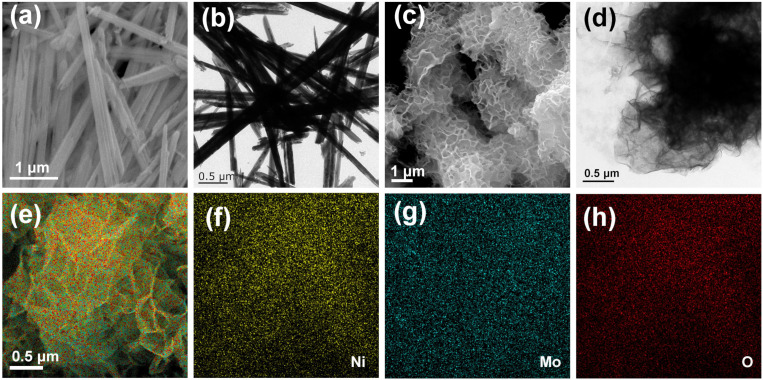
(**a,b**) Morphologies of α-NiMoO_4_ samples. (**c**,**d**) Morphologies of β-NiMoO_4_ samples. (**e**–**h**) Elemental mapping images of β-NiMoO_4_ samples.

**Figure 3:**
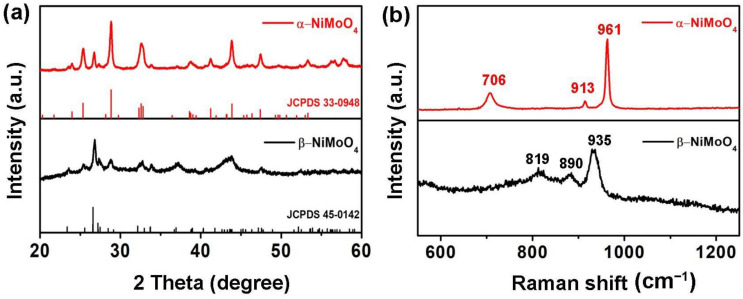
(**a**) XRD patterns of as-synthetic α-NiMoO_4_ and β-NiMoO_4_ samples. (**b**) Raman spectra of as-synthetic α-NiMoO_4_ and β-NiMoO_4_ samples.

SEM and TEM were used to characterize the samples’ morphology, as seen in Figure 2. Figure 2a,b shows the α-NiMoO_4_ sample has a 1D nanorod structure, while the β-NiMoO_4_ sample exhibits a 2D nanosheet structure (Figure 2c,d). The EDS mapping results reveal the β-NiMoO_4_ sample contains homogeneous Ni, Mo, and O elements (Figure 2e–h). XRD patterns of both α-NiMoO_4_ and β-NiMoO_4_ are illustrated in Figure 3a. The main characteristic diffraction peaks in the α-NiMoO_4_ sample are in agreement with monoclinic NiMoO_4_ (JCPDS No.33-0948), while the main characteristic diffraction peaks of the β-NiMoO_4_ sample are consistent with another standard diffraction data of monoclinic NiMoO_4_ (JCPDS No.45-0142). As shown in Figure 3b, the two peaks in the α-NiMoO_4_ sample around 920 cm^−1^ are vibrational peaks of the Mo=O double bond, while the weak peak of 706 cm^−1^ belongs to the vibrational characteristic peak of the Ni-O-Mo bond [43]. In the Raman pattern of the β-NiMoO_4_ sample, three vibrational characteristic peaks belonging to MoO_4_^2−^ can be observed [44]. It can be concluded that α-NiMoO_4_ and β-NiMoO_4_ were synthesized successfully by using the synthesis method in this work.

To investigate the importance of urea for the preparation of β-NiMoO_4_ samples, comparative experiments with different urea concentrations were conducted and the samples’ morphology was analyzed using SEM (Figure S1). Figure S1a depicts the morphology of the urea-free sample, which has a 1D nanorod structure. When 1 M urea was added to the reaction, the morphology of the obtained sample could generally maintain a 1D nanorod structure, but a small number of nanorods dissolved, recrystallized, and gathered together to form a 2D nanosheet structure (Figure S1b). When the urea concentration was increased to 2 M, the morphology of the prepared sample changed to a complete 2D nanosheet structure. This phenomenon suggests that 2 M urea is the key condition for the transition from α-NiMoO_4_ nanorods to β-NiMoO_4_ nanosheets (Figure S1c). However, it is worth noting that samples prepared at 2 M urea do not react uniformly. When the urea concentration was increased to 4 M, the morphology of the resulting product showed a typical 2D nanosheet structure (Figure S1d). Accordingly, the SEM results revealed that the appropriate urea concentration for the synthesis of β-NiMoO_4_ should be 4 M, and the resulting material had an ideal nanosheet morphology. As seen in Figure S2a, the phases of products at various urea concentrations were characterized by XRD. The findings showed that α-NiMoO_4_ had been effectively synthesized as characteristic diffraction peaks of the 0 M and 1 M samples matched monoclinic NiMoO_4_ (JCPDS No.33-0948). The strongest characteristic diffraction peak (2θ = 28.8°) representing α-phase nickel molybdate showed a decreasing trend with increasing urea concentration. The main characteristic diffraction peaks of the 2 M and 4 M samples were in agreement with monoclinic NiMoO_4_ (JCPDS No.45-0142), which confirmed the formation of β-NiMoO_4_. XRD results also confirm that urea significantly influences the phase transition of NiMoO_4_. Specifically, urea is hydrolyzed to form OH^-^ and CO_3_^2−^ in the hydrothermal synthesis process, which can be used as a precipitant to participate in the nucleation and growth of materials. For instance, OH^-^ can neutralize H^+^ in a solution and change the pH of the solution, thus affecting the morphology of materials. CO_3_^2−^ may form compounds with metal ions, which influence the growth rate and orientation of crystals [45]. In addition, pH has a substantial influence on the intermediates generated in the early stages, determining the level of engagement of the topological transformation pathway [46]. Further, the molar ratio (R) of urea and cation also plays a vital role in the nucleation process and is the key to obtaining the self-assembly sequence [47]. As a result, the addition of urea can not only control the nucleation rate and thus affect the morphology and crystalline phase of the material, but it also plays an important role in the molecular stability of metastable transition metal oxides [48]. Figure S2b displays the Raman spectra of samples obtained at different urea concentrations. Characteristic vibrational peaks belonging to α-NiMoO_4_ were observed in the 0 M and 1 M samples, while characteristic vibrational peaks belonging to β-NiMoO_4_ were observed in the 2 M and 4 M samples, aligning with the results obtained from XRD characterization.

Comparative experiments with different reaction times were carried out under the same experimental parameters to investigate the growth mechanism of β-NiMoO_4_. SEM was performed to describe the morphology of the acquired samples with the corresponding results presented in Figure S3. The sample’s morphology showed a uniformly 1D nanorod structure after a 2 h reaction period (Figure S3a). With the increase in the reaction time, the temperature in the autoclave increased steadily, causing the urea to hydrolyze and altering the reaction system’s pH level, which in turn caused the dissolved ions to nucleate and form independent nanoflakes (Figure S3b). In addition, it was found that as the reaction time was further extended, these nanoflakes gradually aggregated to form larger aggregates (Figure S3c,d). Therefore, the SEM results reveal that the appropriate reaction time for the preparation of β-NiMoO_4_ nanosheets is 10 h. As can be seen from Figure S4a, XRD characterization was used to explore the phase differences of the obtained products at different reaction times. The main characteristic diffraction peak of the 2H sample corresponds to the monoclinic NiMoO_4_ (JCPDS No.33-0948), indicating that the prepared product is pure α-NiMoO_4_. With the increase in reaction time, there were not only characteristic diffraction peaks of β-NiMoO_4_ but also trace heterogeneous peaks belonging to α-NiMoO_4_ in the phase of the sample, indicating that the phase of the product was a mixture of α-NiMoO_4_ and β-NiMoO_4_. Moreover, the final product was pure β-NiMoO_4_ when the reaction time was extended to 10 h. In addition, Raman spectra also revealed that with the increase in reaction time, the product gradually changed from pure α-NiMoO_4_ to a mixture of α-NiMoO_4_ and β-NiMoO_4_ and finally formed pure β-NiMoO_4_.The findings from time experiments demonstrate that the structure and crystalline phase of NiMoO_4_ are predominantly influenced by the kinetics of dissolution and growth of precursor in the solution [49].

On the basis of clarifying the suitable urea concentration and reaction time for the preparation of β-NiMoO_4_, the effects of different nickel and molybdenum salt raw materials on the morphology and phase of β-NiMoO_4_ were further explored. The results showed that when nickel nitrate and sodium molybdate were used as reactants, the resulting 2D nanosheet structure showed a spherical shape (Figure S5a). When nickel acetate and sodium molybdate were used as reactants, the resulting product was heteromorphic nanosheets (Figure S5b). In contrast, the 2D nanosheets obtained by the reaction with nickel acetate and ammonium molybdate as reactants exhibited elongation and expansion properties (Figure S5c). The above results indicate that the variation in nickel and molybdenum salt precursors alters the morphology of 2D nanosheets. According to the XRD and Raman patterns of the three samples, the difference between nickel and molybdenum salt raw materials did not cause the change of the phase of products, and β-NiMoO_4_ could be successfully prepared (Figure S6).

### 3.2. Electrochemical Performance Analysis

The samples were examined using a three-electrode system in 3 M KOH to explore the differences in the performance of the electrochemical activities of α-NiMoO_4_ and β-NiMoO_4_. Figure S7a,c show CV curves for two NiMoO_4_ electrodes at scan rates ranging from 5 mV s^−1^ to 200 mV s^−1^. The Faraday reaction between Ni^2+^/Ni^3+^ is responsible for the redox peaks in CV plots [50]. Figure S7b,d show GCD curves of two NiMoO_4_ electrodes at current densities ranging from 0.5 to 20 A/g. The symmetrical charging and discharging curves indicate that their electrochemical reversibility performs well.

**Figure 4:**
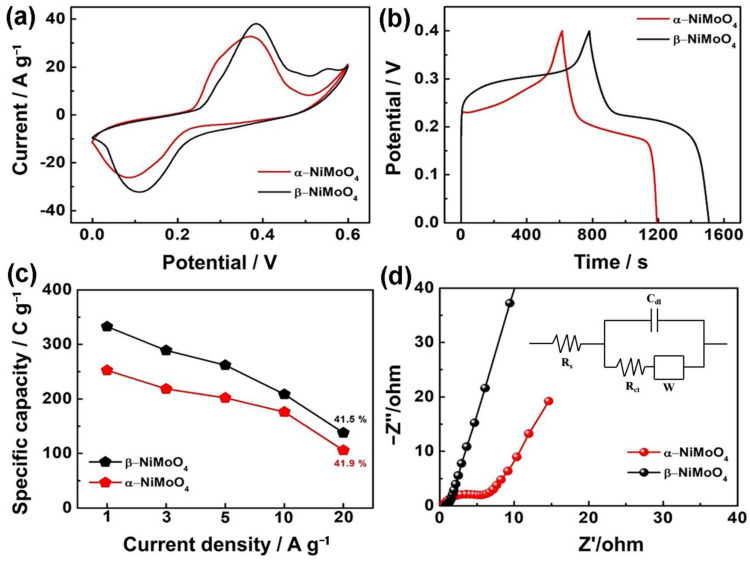
Comparison of electrochemical performance between two NiMoO_4_ electrodes: (**a**) CV test results at 20 mV/s, (**b**) GCD test results at 0.5 A/g, (**c**) rate performance, and (**d**) Nyquist plots.

The CV curves of two NiMoO_4_ electrodes at 20 mV/s and a voltage window range of 0~0.6 V are shown in Figure 4a. The β-NiMoO_4_ electrode exhibits greater integration area compared to the α-NiMoO_4_ electrode, suggesting the β-NiMoO_4_ electrode possesses superior specific capacity relative to that of the α-NiMoO_4_ electrode. The 2D nanosheet structure has a larger active specific surface area, which is favorable for rapid electrolyte intercalation/diffusion. By comparing the GCD curves of two NiMoO_4_ electrodes at 0.5 A/g (Figure 4b), the discharge time of the β-NiMoO_4_ electrode was significantly longer compared to that of the α-NiMoO_4_ electrode. This outcome aligns with the analysis of the integral regions of CV curves, thereby reinforcing the conclusion that the specific capacity of the β-NiMoO_4_ electrode surpasses that of the α-NiMoO_4_ electrode. Figure 4c displays the rate performance of two NiMoO_4_ electrodes, which have a capacity retention of about 41.5% at 20 A/g. The electrochemical properties of two NiMoO_4_ electrodes were further investigated by EIS. A quasi-semicircle in the high-frequency zone and a straight line in the low-frequency region make up the Nyquist plot. The rapid diffusion and adsorption behavior of ions on the electrode surface is revealed by the slope of the line in the low-frequency zone. The equivalent series resistance (R_s_) is represented by the semicircle’s starting point intersecting the horizontal axis. As shown in Figure 4d, the slope of the linear part of the β-NiMoO_4_ electrode is steeper compared to that of the α-NiMoO_4_ electrode, signifying faster ion diffusion on the surface of the β-NiMoO_4_ electrode [51]. For a high-frequency region, the β-NiMoO_4_ electrode possesses an R_s_ value of 0.41 Ω, which is superior to the α-NiMoO_4_ (0.78 Ω) electrode. In addition, the β-NiMoO_4_ electrode shows a capacity retention of 77.6% following 5000 charge-discharge cycles under 5 A/g current density, which is improved compared to that of the α-NiMoO_4_ electrode (Figure S8). The electrochemical performance of some metal-oxide-based materials is summarized along with our work in Table S1. It indicates that our prepared β-NiMoO_4_ has advantages over other materials.

**Figure 5:**
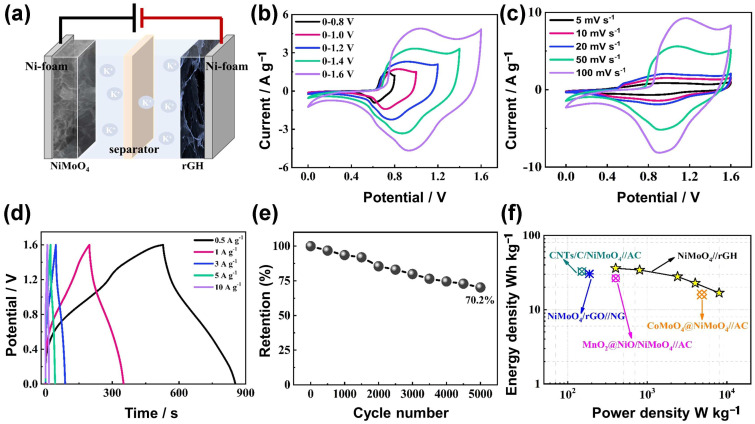
(**a**) Assembly diagram of NiMoO_4_//rGH device. (**b**) Suitable voltage window test results of NiMoO_4_//rGH device at 20 mV/s. (**c**) CV and (**d**) GCD results of the NiMoO_4_//rGH device. (**e**) Cycling performance of as-fabricated NiMoO_4_//rGH ASC at 5 A/g. (**f**) Ragone curves of as-fabricated NiMoO_4_//rGH ASC.

To explore the potential practical applications of NiMoO_4_ materials, an ASC was assembled by using reduced graphene oxide hydrogel (rGH) as a negative electrode and β-NiMoO_4_ as a positive electrode. The morphology of rGH and its electrochemical properties can be seen in Figures S9 and S10. The assembly schematic of the NiMoO_4_//rGH device is illustrated in Figure 5a, and a suitable potential window of 1.6 V was determined from CV curves across various voltage ranges (Figure 5b). No obvious changes were observed in the form of the ASC device’s CV curves at varied scan rates, demonstrating its good kinetic characteristics (Figure 5c). According to the GCD curves of the device (Figure 5d), the specific capacities of NiMoO_4_//rGH device are 162.4, 153.8, 125.4, 102.4 and 75.7 C/g at 0.5, 1, 3, 5 and 10 A/g, respectively. An EIS test was also conducted to characterize the resistance of NiMoO_4_//rGH device (Figure S11). As can be seen from Figure 5e, the NiMoO_4_//rGH device shows a retention of 70.2% following 5000 charge-discharge cycles under 5 A/g. The energy and power densities of NiMoO_4_//rGH device were determined to be 36.1 W h kg^−1^ at 399.9 W kg^−1^ (detailed formulas in Supplementary Materials). Ragone plots (Figure 5f) demonstrate that the performance of the as-assembled NiMoO_4_//rGH device surpasses that of other Ni-Mo oxide devices as reported in the literature (Table S2) [52,53,54,55]. The enhanced performance indicates NiMoO_4_ has a wide variety of potential applications.

The β-NiMoO_4_ electrode has better electrochemical performance than the α-NiMoO_4_ electrode, which can be summarized for the following reasons. The electrochemical performance of β-NiMoO_4_ is enhanced due to the substantial increase in active sites involved in the electrochemical processes resulting from the change from 1D nanorod structure to 2D nanosheet structure. The electron state of β-NiMoO_4_ is more localized than that of α-NiMoO_4_, and its localization near the Fermi level and enhanced density of states (DOS) give β-NiMoO_4_ a higher specific capacity than α-NiMoO_4_ [56]. Additionally, Mo^6+^ ions in α-NiMoO_4_ are coordinated octahedrally, whereas they adopt tetrahedral coordination in β-NiMoO_4_. In a β-phase tetrahedral environment, Mo^6+^ ions are unsaturated coordination. The presence of a partially empty 4d orbital near the Mo atom helps store more charge, which makes β-NiMoO_4_ more electrochemically active [57]. In summary, the β-NiMoO_4_ electrode exhibits improved specific capacity and cycling performance compared with the α-NiMoO_4_ electrode.

## 4. Conclusions

To sum up, a simple approach was introduced to achieve exact control of NiMoO_4_. The structural transition from α-NiMoO_4_ nanorods to β-NiMoO_4_ nanosheets leads to an increased number of active sites, thus improving its electrochemical activity. As expected, the partially vacant 4d orbitals near molybdenum atoms in β-NiMoO_4_ help to store more charges, endowing the material with an enhanced specific capacity of 332.8 C/g along with good cycling stability. Moreover, the NiMoO_4_//rGH device exhibits an energy density of 36.1 W h kg^−1^ at a power density of 399.9 W kg^−1^. The findings might help guide the optimal design of materials with customized nanostructures in other energy storage applications.

## Data Availability

Data are contained within the article and Supplementary Materials.

## Supplementary Materials

The following supporting information can be downloaded at: https://www.mdpi.com/article/10.3390/nano14221798/s1, Figure S1: Morphologies of the samples acquired at (a) 0 M, (b) 1 M, (c) 2 M, (d) 4 M urea; Figure S2: (a) Comparison of XRD results of 0–4 M urea reaction products. (b) Comparison of Raman results of 0–4 M urea reaction products; Figure S3: Morphologies of the samples acquired at (a) 2 h, (b) 4 h, (c) 6 h, (d) 10 h; Figure S4: (a) Comparison of XRD test results of time experiment samples. (b) Comparison of Raman test results of time experiment samples; Figure S5: Morphologies of the samples after reaction of (a) nickel nitrate and sodium molybdate, (b) nickel acetate and sodium molybdate, (c) nickel acetate and ammonium molybdate; Figure S6: (a) Comparison of XRD patterns of the samples after reaction of ① nickel nitrate and sodium molybdate, ② nickel acetate and sodium molybdate, ③ nickel acetate and ammonium molybdate. (b) Comparison of Raman spectra of above three samples; Figure S7: (a) CV curves of α-NiMoO_4_ electrode, (b) GCD curves of α-NiMoO_4_ electrode, (c) CV curves of β-NiMoO_4_ electrode, (d) GCD curves of β-NiMoO_4_ electrode; Figure S8: Long-term cycling stability of two NiMoO_4_ electrodes; Figure S9: (a) CV curves of rGH electrode at different scan rates. (b) GCD curves of rGH electrode at different current densities; Figure S10: (a) Photograph of as-prepared rGH. (b) SEM image of 3D rGH; Figure S11: Nyquist plot of the NiMoO_4_//rGH device; Table S1: Comparison of the performance of various metal oxide-based electrodes reported in recent years.; Table S2: Comparison of electrochemical performance of our assembled NiMoO_4_//rGH device with other reported Ni-Mo oxide devices; Calculation equations of two and three electrodes. References [45,52,58,59,60,61,62,63,64,65,66,67,68,69] are included in the Supplementary Materials.
