# Supplementary material for: Synergistic Regulation of Phase and Nanostructure of Nickel Molybdate for Enhanced Supercapacitor Performance
Source: Nanomaterials (Basel). 2024 Nov 8;14(22):1798. doi: 10.3390/nano14221798 (PMC11597826; doi:10.3390/nano14221798)
Supplement: Supplementary file 1 [file nanomaterials-14-01798-s001.zip › nanomaterials-3282898-supplementary.pdf]

Supplementary Materials

Synergistic regulation of phase and nanostructure of nickel molybdate for enhanced supercapacitor performance

Yining Wang [1, 2]*, Yuhan Cui [1], Yue Song [1] and Chen Zhou [2]

[1] School of Chemistry and Environmental Engineering, Changchun University of Science and Technology, Changchun130022, China;

[2] Jilin Provincial International Joint Research Center of Photo-functional Materials and Chemistry, Changchun130022, China;

* Correspondence: yiningwang@cust.edu.cn

Section 1 The other characterizations and electrochemical measurements of contrasted samples

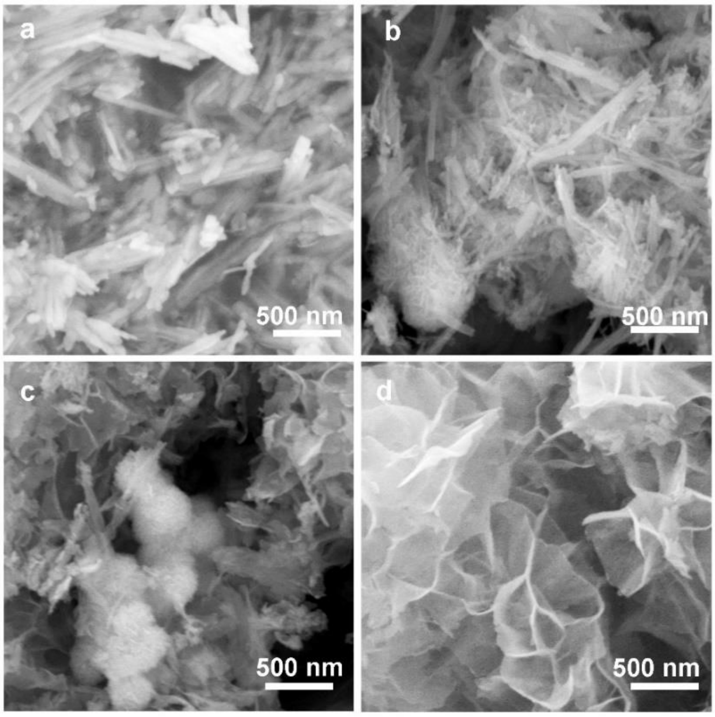

Figure S1. Morphologies of the samples acquired at (a) 0 M, (b) 1 M, (c) 2 M, (d) 4 M urea.

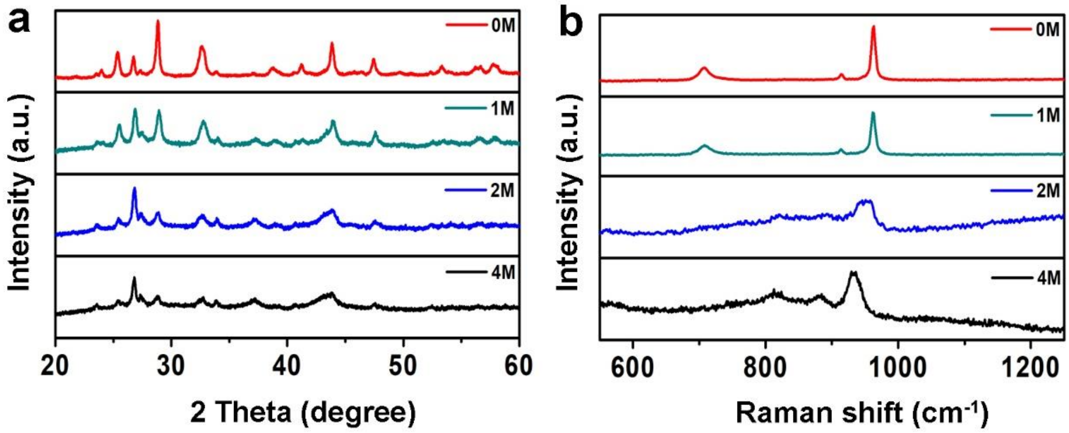

Figure S2. (a) Comparison of XRD results of 0 - 4M urea reaction products. (b) Comparison of Raman results of 0 - 4M urea reaction products.

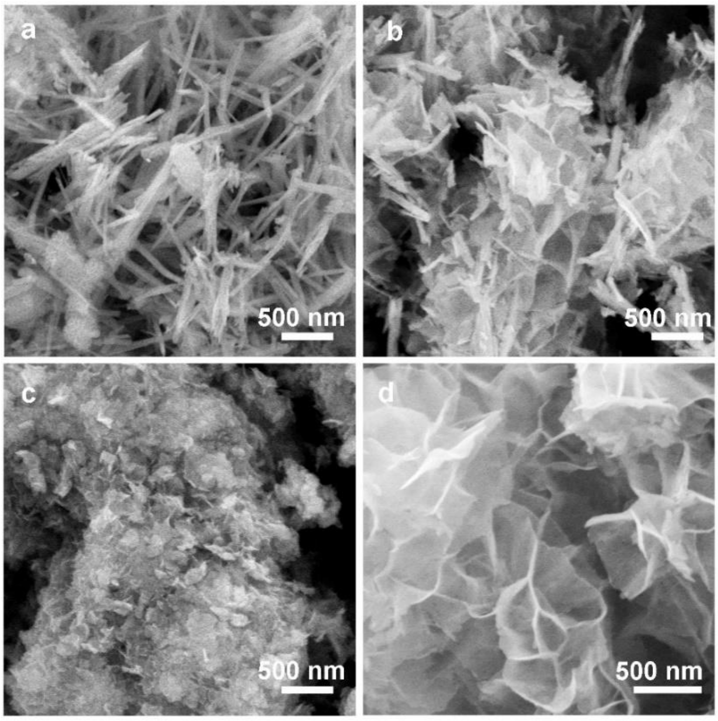

Figure S3. Morphologies of the samples acquired at (a) 2 h, (b) 4 h, (c) 6 h, (d) 10 h.

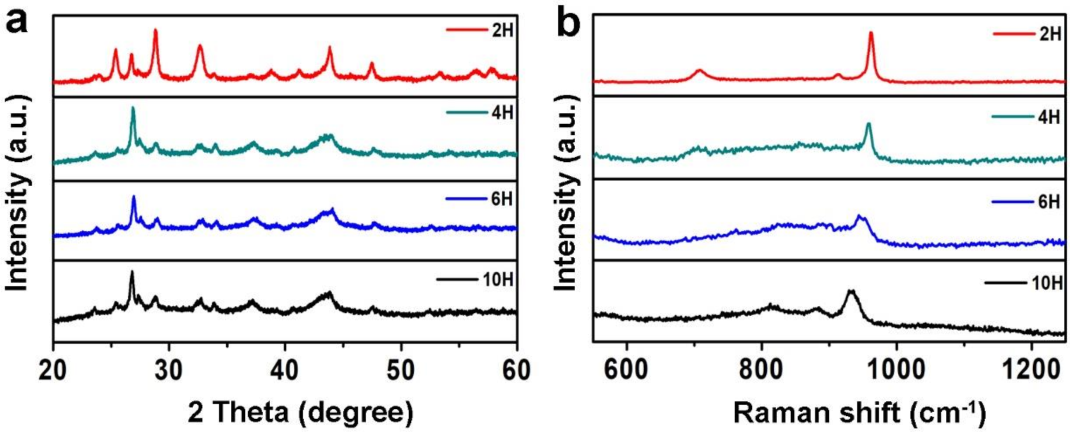

Figure S4. (a) Comparison of XRD test results of time experiment samples. (b) Comparison of Raman test results of time experiment samples.

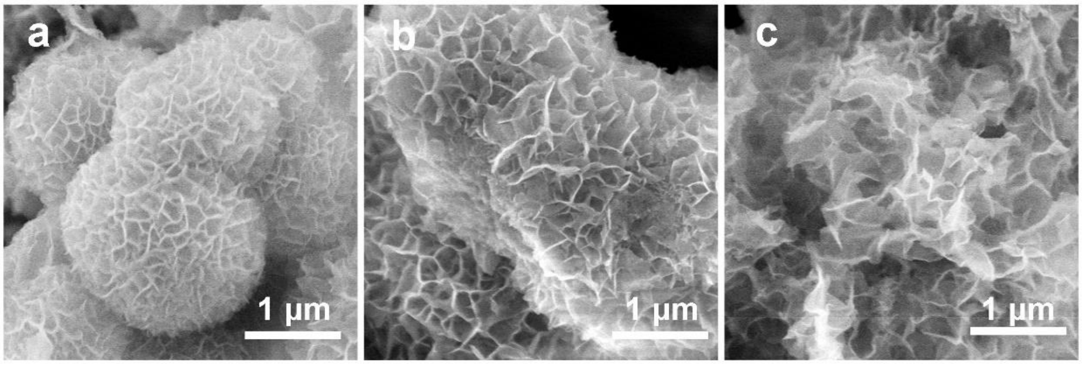

Figure S5. Morphologies of the samples after reaction of (a) nickel nitrate and sodium molybdate, (b) nickel acetate and sodium molybdate, (c) nickel acetate and ammonium molybdate.

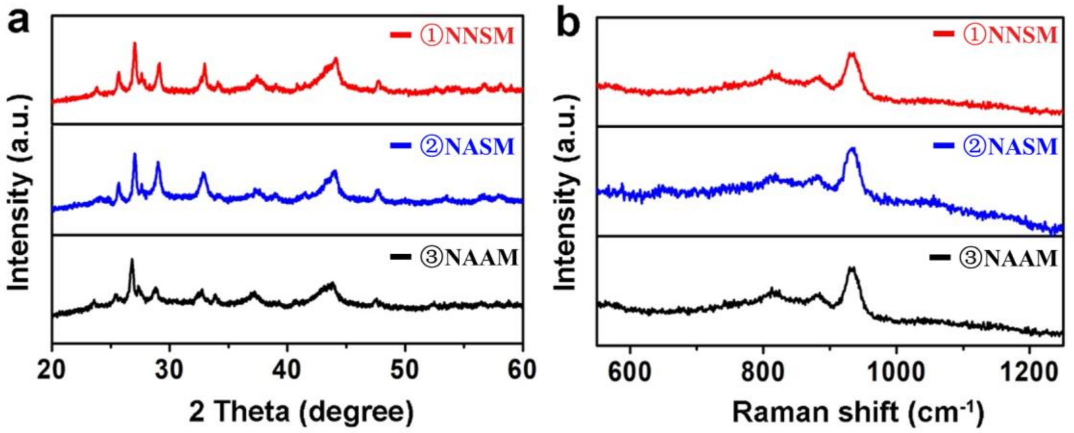

Figure S6. (a) Comparison of XRD patterns of the samples after reaction of ① nickel nitrate and sodium molybdate, ② nickel acetate and sodium molybdate, ③ nickel acetate and ammonium molybdate. (b) Comparison of Raman spectra of above three samples.

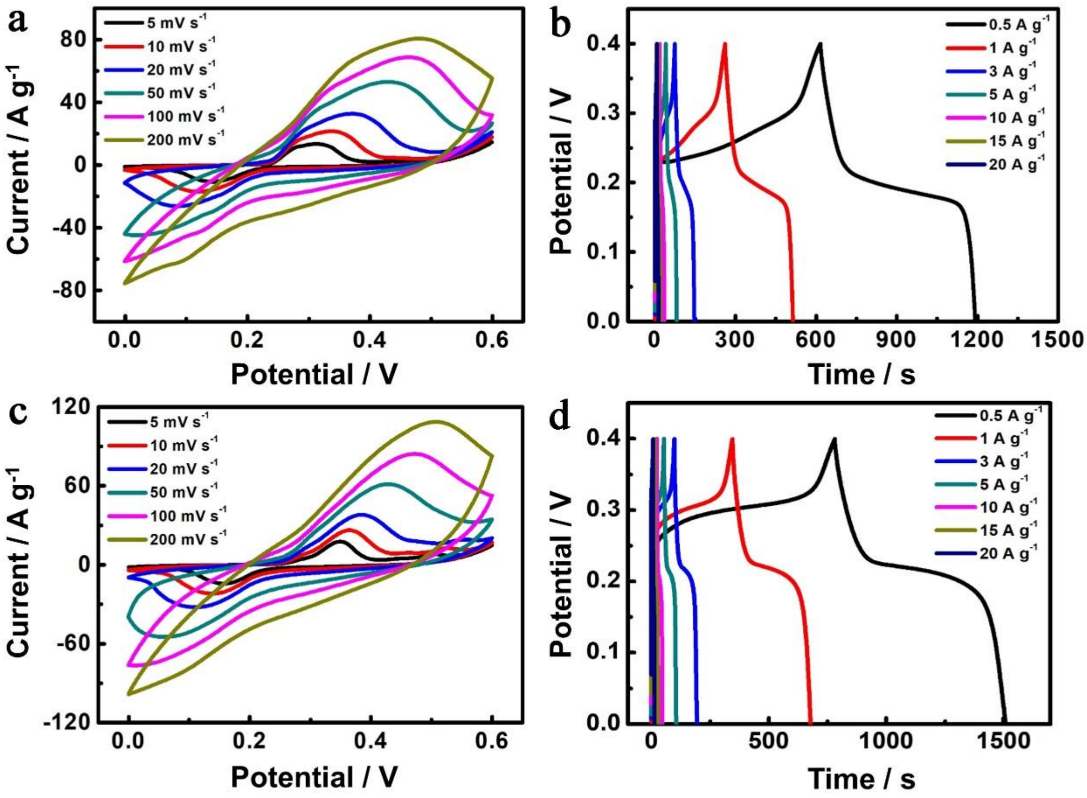

Figure S7. (a) CV curves of α-$NiMoO_4$ electrode, (b) GCD curves of α-$NiMoO_4$ electrode, (c) CV curves of β-$NiMoO_4$ electrode, (d) GCD curves of β-$NiMoO_4$ electrode.

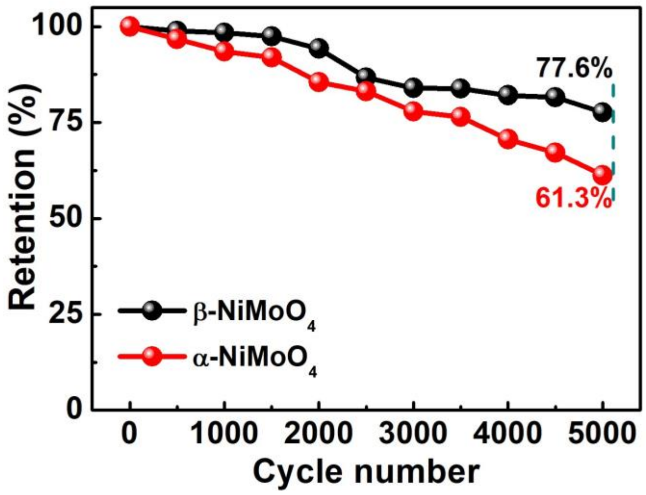

Figure S8. Long-term cycling stability of two $NiMoO_4$ electrodes.

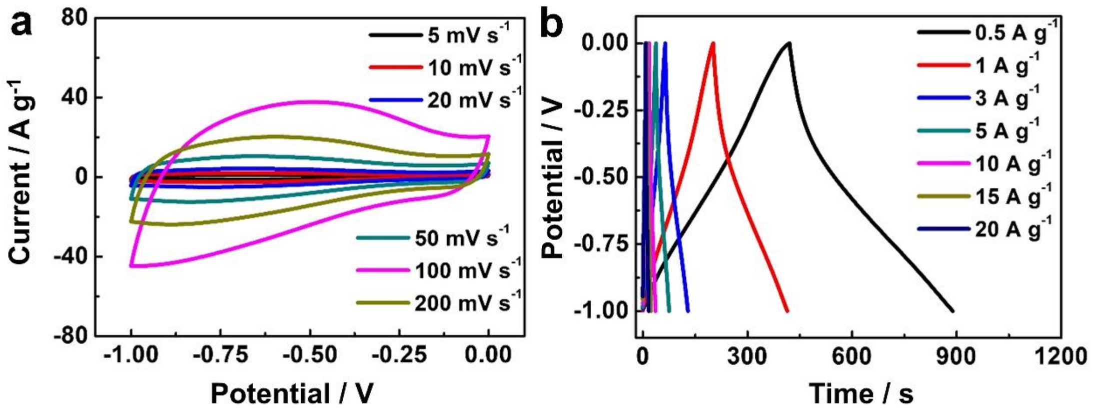

Figure S9. (a) CV curves of rGH electrode at different scan rates. (b) GCD curves of rGH electrode at different current densities.

The electrochemical performance of the rGH was evaluated by a standard three-electrode system. Figure S9a shows the CV curves of the rGH electrode at different scan rates, and its current response increases with increasing scan rate. Figure S9b displays the GCD curves of the rGH electrode at different current densities. The specific capacitance of the rGH electrode calculated from the GCD curves is 224.2 F/g at 0.5 A/g.

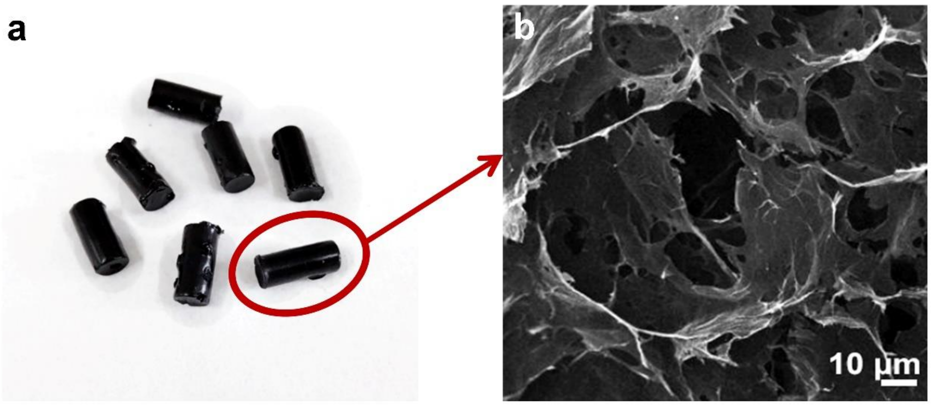

Figure S10. (a) Photograph of as-prepared rGH. (b) SEM image of 3D rGH.

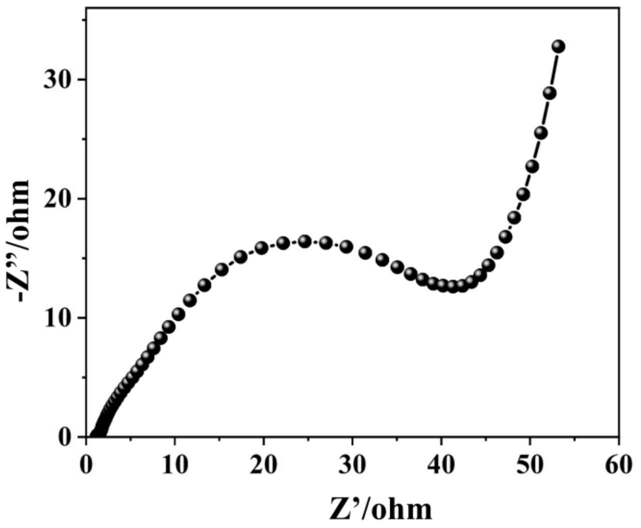

Figure S11. Nyquist plot of the $NiMoO_4$//rGH device.

Table S1 Comparison of the performance of various metal oxide-based electrodes reported in recent years.

Material	Electrolyte	Specific capacitance ($F\ g^{-1}$)	retention (%) / cycle number	Ref.
NiO	1 M KOH	74 at 10 mA g^{-1}	75/500	S1
NiO	2 M KOH	246 at 0.5 mA cm^{-2}	92/500	S2
$NiCo_2O_4$	3 M KOH	734 at 1 A g^{-1}	85/1000	S3
$NiMn_2O_4$	1 M KOH	219 at 1 A g^{-1}	90.6/3000	S4
$NiMoO_4$	1 M KOH	616 at 1 A g^{-1}	83.9/5000	S5
$CoMoO_4$-$NiMoO_4$	3 M KOH	751 at 1 A g^{-1}	94/2000	S6
C/$NiMoO_4$	2 M NaOH	268.8 at 1 A g^{-1}	88.4/2000	S7
$NiMoO_4$/graphene	2 M KOH	670 at 0.3 A g^{-1}	88/3000	S8
$NiMoO_4$/rGO	6 M KOH	819 at 5 A g^{-1}	81.1/1000	S9
β-$NiMoO_4$	3 M KOH	833 at 1 A g^{-1}	77.6/5000	This work

Table S2. Comparison of electrochemical performance of our assembled $NiMoO_4$//rGH device with other reported Ni-Mo oxide devices.

**Material**	**Specific Current ($A\ g^{-1}$)**	**Specific Capacitance ($F\ g^{-1}$)**	**retention (%) / cycle number**	**Energy density (Wh kg^{-1})**	**Power density (W kg^{-1})**	**Ref.**
$NiMoO_4$/rGO//NG	0.5	84	84.6/1000	30.3	187	47
CNTs/C/$NiMoO_4$//AC	0.2	69.5	96.5/1500	32.6	150	48
$CoMoO_4$@$NiMoO_4$//AC	--	--	99/3000	28.7	267	49
MnO_2@NiO/$NiMoO_4$//AC	0.5	74.4	80/10000	26.5	401	50
$CoMoO_4$-$NiMoO_4$//AC	--	--	--	30.86	270	S6
α-$NiMoO_4$//rGO	0.5	73.3	85/2000	12.31	274.91	S10
$NiCo_2O_4$@ $NiMoO_4$//AC	5 mA/cm^2	61.7	85.2/3000	21.7	157	S11
50 NiMo-HCNF//AC	0.5	135 C g^{-1}	92/3000	30	403	S12
$NiMoO_4$//rGH	0.5	162.4	70.2/5000	36.09	399.98	This work

Section 2 Calculation equations of two and three electrodes

Usually, the specific capacitance of samples was evaluated from the galvanostatic charge-discharge curves according to the following equation (1) [S13].

$$C = \frac{I \times \Delta t}{m \times \Delta V} \tag{1}$$

where C (F g^{-1}) is the specific capacitance, I (A) represents the constant discharge current, Δt (s) is the discharge time, m (g) is the mass of the active material and ΔV (V) is the potential window.

The specific capacity of samples was evaluated from the galvanostatic charge-discharge curves according to the following equation (2):

$$Q = \frac{I \times \Delta t}{m} \tag{2}$$

where Q (C g^{-1}) is the specific capacity, the definitions of I, Δt and m are the same as those in equation (1).

For two-electrode asymmetric supercapacitor devices, the mass ratio between the positive and negative electrode was obtained according to the following equation (3). And the energy density E (Wh kg^{-1}) and power density P (W kg^{-1}) of the ASC are calculated based on the equations (4) - (5) [S14].

$$\frac{m+}{m-} = \frac{C_{-} \times \Delta V_{-}}{Q_{+}} \tag{3}$$

$$E = \frac{Q \times \Delta V}{2 \times 3.6} \tag{4}$$

$$P = \frac{E \times 3600}{\Delta t} \tag{5}$$

where C (F g^{-1}) is the specific capacitance, ΔV is the potential drop during the discharge process, Q (C g^{-1}) is the specific capacity and Δt is the discharge time.

References

1. S. Vijayakumar, S. Nagamuthu and G. Muralidharan, *ACS Appl. Mater. Interfaces*, 2013, **5**, 2188-2196.

2. S. D. Dhas, P. S. Maldar, M. D. Patil, A. B. Nagare, M. R. Waikar, R. G. Sonkawade and A. V. Moholkar, *Vacuum*, 2020, **181**, 109646.

3. Z. Wu, Y. Zhu and X. Ji, *Journal of Materials Chemistry A*, 2014, **2**, 14759-14772.

4. S. Prakash and G. Paruthimal Kalaignan, *Colloids and Surfaces A: Physicochemical and Engineering Aspects*, 2021, **611**, 125875.

5. P. Dhandapani, P. K. Nayak and A. Maruthapillai, *Electrochim. Acta*, 2024, **491**, 144260.

6. Q. Yang and S.-Y. Lin, *RSC Advances*, 2016, **6**, 10520-10526.

7. C. Wei, Y. Huang, J. Yan, X. Chen and X. Zhang, *Ceram. Int.*, 2016, **42**, 15694-15700.

8. E. R. Ezeigwe, P. S. Khiew, C. W. Siong, I. Kong and M. T. T. Tan, *Ceram. Int.*, 2017, **43**, 13772-13780.

9. T. Liu, H. Chai, D. Jia, Y. Su, T. Wang and W. Zhou, *Electrochim. Acta*, 2015, **180**, 998-1006.

10. P. R. Jothi, K. Shanthi, R. R. Salunkhe, M. Pramanik, V. Malgras, S. M. Alshehri and Y. Yamauchi, *Eur. J. Inorg. Chem.*, 2015, **2015**, 3694-3699.

11. D. Cheng, Y. Yang, J. Xie, C. Fang, G. Zhang and J. Xiong, *Journal of Materials Chemistry A*, 2015, **3**, 14348-14357.

12. V. S. Budhiraju, R. Kumar, A. Sharma and S. Sivakumar, *Electrochim. Acta*, 2017, **238**, 337-348.

13. X. Wang, H. Ding, W. Luo, Y. Yu, Q. Chen, B. Luo, M. Xie and X. Guo, *EcoEnergy*, 2023, **1**, 448-459.

14. S. M. Youssry, M. A. Elkodous, R. Kumar, G. Kawamura, W. K. Tan and A. Matsuda, *Electrochim. Acta*, 2023, **463**, 142814.
